# Navigating Continuous Glucose Monitoring Adoption: Insights From Hispanic Adults With Insulin‐Treated Type 2 Diabetes

**DOI:** 10.1155/jdr/7474846

**Published:** 2026-04-24

**Authors:** Diana Soliman, Giuliana Arevalo, Rodolfo J. Galindo, Benjamin Churba, Keilecia G. Malden, Daniel E. Jimenez, Francisco J. Pasquel, David A. Baidal, Ashby F. Walker

**Affiliations:** ^1^ Division of Endocrinology, Diabetes and Metabolism, Department of Medicine, University of Miami Leonard M. Miller School of Medicine, Miami, Florida, USA, miami.edu; ^2^ Department of Health Services Research, Management and Policy, University of Florida, Gainesville, Florida, USA, ufl.edu; ^3^ Department of Psychiatry and Behavioral Sciences, University of Miami Miller School of Medicine, Miami, Florida, USA, miami.edu; ^4^ Division of Endocrinology, Department of Medicine, Emory University School of Medicine, Atlanta, Georgia, USA, emory.edu

**Keywords:** continuous glucose monitoring (CGM), hispanic adults, social–ecological model, type 2 diabetes mellitus (T2DM)

## Abstract

**Objective:**

Hispanic individuals are disproportionately affected by type 2 diabetes (T2D) and face access limitations in diabetes technology utilization. Despite proven benefits, continuous glucose monitoring (CGM) use remains low among Hispanic adults with insulin‐treated T2D. This study used a social–ecological model (SEM) framework to identify societal/policy, interpersonal/community, and individual level barriers to CGM access and adoption within this population.

**Methods:**

Two focus groups were conducted in Spanish at the University of Miami (UM) with Hispanic adults (*n* = 16) with T2D. Inclusion criteria were age ≥18 years, HbA1c ≥8%, ≥1 daily insulin injection, and no CGM use in the past 2 years. The initial focus group was conducted to identify barriers to CGM initiation. At the end of the focus group, participants were provided with real‐time CGMs for 30 days of use. After completing the wear period, the same participants returned for a follow‐up focus group to explore factors related to CGM adoption and acceptance. Focus groups were analyzed using a thematic analysis, systematically coding and categorizing key concepts and ideas expressed by participants.

**Results:**

The mean age was 59.68 ± 13.37 years, and baseline HbA1c was 9.78 ± 1.14. Key barriers included high device costs, limited insurance coverage, alarm fatigue, and lack of culturally concordant provider guidance. Facilitators for CGM use included manufacturer discounts, peer modeling, and the desire to avoid fingersticks. After a 30‐day CGM trial, most participants reported improved dietary awareness, glycemic control, and a strong desire for continued use.

**Conclusion:**

This pilot study provides novel insights into the experiences of the Hispanic population and highlights the complex interplay of societal, interpersonal, and individual‐level factors influencing CGM use. Tailored interventions addressing these barriers are crucial for improving CGM utilization in this population.

## 1. Introduction

Type 2 diabetes (T2D) disproportionately affects Hispanic adults in the United States, with a prevalence of 22.1% compared to 12.1% in the non‐Hispanic White (NHW) population [1]. In addition, Hispanic adults with T2D experience worse glycemic control and increased risk of diabetes‐related complications compared to NHW individuals [2–4]. Continuous glucose monitoring (CGM), which provides real‐time measurements of interstitial glucose levels, allows patients to track glucose trends and make informed decisions about diet, physical activity, and medication dosing [5, 6]. In people with T2D treated with insulin, CGM has been shown to improve glycemic control and reduce the risk of hypoglycemia‐ and hyperglycemia‐related hospital admissions [7, 8]. The American Diabetes Association (ADA) and the American Association of Clinical Endocrinology (AACE) strongly recommend CGM use for all insulin‐treated individuals with diabetes [9, 10].

Despite these benefits, CGM utilization remains limited among Hispanic adults with diabetes [6, 11–13]. Few studies have examined barriers to diabetes technology use among this vulnerable population [12–14]. While research has identified racial, ethnic, insurance‐mediated, and provider bias as barriers to technology adoption in type 1 diabetes (T1D) [15–20], there is a gap in understanding barriers to CGM use in insulin‐treated T2D populations, especially among Hispanic patients who face unique cultural, linguistic, and socioeconomic barriers.

Identification of the specific challenges preventing Hispanic individuals from using CGM technology is important for developing targeted interventions. This pilot study used a social–ecological model (SEM) to explicate barriers and facilitators to CGM use among Hispanic individuals with insulin‐treated T2D at the societal/policy, interpersonal/community, and individual levels by utilizing focus groups before and after CGM use for 30 days. We also aimed to compare the factors influencing CGM acceptance and adoption between Hispanic adults <65 years and those ≥65 years.

## 2. Methods

This qualitative study used focus groups to explore barriers to CGM use among Hispanic adults with T2D using insulin. Focus group domains and questions were developed using the conceptual framework of the social–ecological model (SEM) [21] and elements of the Consolidated Framework for Implementation Research [22]. A focus group script was developed and reviewed by six content experts, including four endocrinologists with 5–20 years of clinical and research experience in adult endocrinology, a psychologist and qualitative researcher with over 20 years of experience, and a public health researcher with more than 10 years of experience in focus group methodology. A pre‐focus group survey was also developed using REDCap and used to capture overall participant demographics, baseline CGM knowledge, and aspects of current diabetes care. Focus groups were conducted in Spanish at the University of Miami (UM) from winter of 2023 to spring of 2024 and took place twice: once prior to using CGMs and another session after participants had an opportunity to use CGMs. Four focus group sessions were conducted in total: two pre‐CGM sessions and two post‐CGM sessions. Participants attended sessions according to their age subgroup and returned with the same group for the follow‐up session. All research protocols and consent processes were approved by the institutional review board at UM.

### 2.1. Participants

Hispanic adults with insulin‐treated T2D were recruited from the UM Endocrinology clinic between December 2023 and February 2024. Two focus groups were conducted by age subgroup: a “young adult” group (18–65 years; *n* = 11) and an elderly group (≥65 years; *n* = 5). Each group participated in two sessions conducted in Spanish—one prior to CGM use and one after participants had an opportunity to use CGMs—resulting in four focus group sessions in total. Participants remained in the same group for both sessions. Inclusion criteria were age ≥18 years, Hispanic or Latino/a ethnicity, diagnosis of T2D for at least 6 months, use of at least 1 or more injections of insulin per day, hemoglobin A1*c* ≥8% at enrollment, and ability to speak and understand the Spanish language.

### 2.2. Focus Group Protocol

Informed consent was obtained and documented from all participants in person via paper consent prior to the first focus group. Participants completed an in‐person written baseline survey prior to the first focus group, collecting demographic information and diabetes‐related care questions. Focus groups were conducted in Spanish, which lasted 1 h, and followed a semi‐structured format. They were led by a trained Spanish‐speaking moderator and audio‐recorded for transcription. A multidisciplinary team comprised of endocrinologists, a clinical psychologist, and a qualitative research expert developed the focus group protocol. The script included general topic domains and specific discussion prompts designed to explore participants’ perceptions, knowledge, and experiences related to CGM technology (Table 1). Participants were compensated $100 for their time filling out pre‐focus group surveys, participating in each focus group session, and covering any associated transportation costs.

**Table 1:** Focus group sample questions.

Focus group questions before continuous glucose monitor use
If you know about continuous glucose monitors, where do you go for information about them?
How do you envision the continuous glucose monitor affecting your daily life, activities, and routines?
What are some of the ways you think a continuous glucose monitor might improve your life or experience with diabetes management?
Are there any specific challenges or fears you have regarding wearing a glucose monitor continuously?
What would you say are some of the major obstacles to obtaining a continuous glucose monitor?

**Focus group questions after continuous glucose monitor use**

Can you describe your overall experience using the continuous glucose monitor for 30 days?
What aspects of your daily life or routines were affected by the continuous glucose monitor?
Were there any specific challenges you encountered while using the continuous glucose monitor, and how did you address them?
What benefits did you notice in terms of managing your diabetes or overall well‐being while wearing the continuous glucose monitor?
Are there any specific fears you have regarding wearing a glucose monitor continuously?

### 2.3. CGM Wear Period

Following the initial focus group, participants were provided with a 30‐day supply of CGM sensors (Dexcom G7). A trained study member provided education on CGM placement, mobile application installation and operation, and sensor removal and disposal. The same participants returned after the wear period for a follow‐up focus group aimed at exploring factors related to CGM acceptance. Eight weeks after the completion of the second focus group, a structured follow‐up assessment was conducted. The research team contacted each participant via telephone to determine whether participants had successfully obtained a CGM through their healthcare provider and insurance benefits.

### 2.4. Data Analysis

Focus group audio recordings were transcribed and translated by Datagain Inc., an external qualitative analysis vendor, and a thematic analysis was conducted using two coders using a method of constant comparison associated with grounded theory [23]. This approach involved iteratively comparing data segments to identify recurring patterns and themes, allowing for the emergence of key concepts directly from the participants’ narratives. In addition to the external thematic analysis conducted by Datagain Inc., subsequent content analysis was conducted by two coders of all quotes from participants related to barriers to CGM use. Each coder independently analyzed external thematic analysis and transcripts. The SEM served as the framework for the coding scheme. This model facilitates the identification of barriers across multiple levels, including individual, interpersonal/community, and societal/policy levels. To ensure coding reliability, inter‐rater reliability (IRR) was assessed using percentage agreement between the two coders and Cohen’s kappa statistic [24]. The study protocol was approved by the University of Miami Institutional Review Board.

## 3. Results

### 3.1. Survey Data

A total of *n* = 16 adults with T2D participated in focus groups. The mean age was 59.68 ± 13.37 years, and baseline HbA1c was 9.78 ± 1.14. There were 11 participants with the 18‐to‐65‐year age group and 5 participants in the ≥65 years group. Table 2 presents participant demographics stratified by whether they independently obtained a CGM by the 8‐week follow‐up call. Overall, more than half of participants reported an income of less than $35,000 per year. Participants reported a wide range of countries of origin including, Cuba (*n* = 6), Dominican Republic (*n* = 3), Nicaragua (*n* = 1), Peru (*n* = 1), Venezuela (*n* = 1), and the United States (*n* = 4, including 1 participant from the United States territory of Puerto Rico). The mean number of years living in the United States was 35.62 ± 19.52 years. All participants reported having T2D for more than 5 years, and most (62.5%) were on insulin for more than 5 years. In the single literacy item, 86% of participants reported “never” needing assistance with reading instructions, pamphlets, or other written materials from their doctor or pharmacy.

**Table 2:** Baseline demographic characteristics stratified by continuous glucose monitor (CGM) uptake at 8‐week follow‐up.

Characteristic	Obtained CGM (*n* = 11)	Did not obtain CGM (*n* = 5)
Age in years, mean (SD)	58.6 (15.6)	62.2 (6.9)
Sex, *n* (%)		
Male	5 (45.5)	1 (20.0)
Female	6 (54.5)	4 (80.0)
Self‐reported race, *n* (%)		
White	9 (81.8)	5 (100.0)
Black (or African–American)	2 (18.2)	–
Country of origin, *n* (%)		
Cuba	2 (18.2)	4 (80.0)
Dominican Republic	3 (27.3)	–
Nicaragua	1 (9.1)	–
Peru	1 (9.1)	–
Venezuela	1 (9.1)	–
U.S. (including Puerto Rico)	3 (27.3)	1 (20.0)
Years living in the USA, mean (SD)	37.5 (20.8)	31.6 (18.0)
Education level, *n* (%)		
Less than high school	1 (9.1)	1 (20.0)
High school diploma	2 (18.2)	4 (80.0)
Some college	4 (36.4)	–
College degree (associate’s, bachelor’s, or equivalent)	4 (36.4)	–
Yearly income ($), *n* (%)		
<35,000	3 (27.3)	4 (80.0)
35,000 to <50,000	1 (9.1)	1 (20.0)
50,000 to <75,000	3 (27.3)	–
Do not wish to provide	4 (36.4)	–
Insurance, *n* (%)		
Private	4 (36.4)	2 (40.0)
Medicare	1 (9.1)	2 (40.0)
Medicaid	1 (9.1)	1 (20.0)
Other/do not wish to provide	5 (45.5)	–
HbA1c, mean (SD)	9.9 (1.3)	9.6 (0.7)
Time of insulin use, *n* (%)		
<6 months	1 (9.1)	–
1–2 years	2 (18.2)	–
2–5 years	2 (18.2)	1 (20.0)
>5 years	6 (54.5)	4 (80.0)
Type of insulin used, *n* (%)		
Basal only	6 (54.5)	2 (40.0)
Basal + bolus	5 (45.5)	3 (60.0)
Insulin injection times per day, *n* (%)		
1 time/day	6 (54.5)	1 (20.0)
2 times/day	2 (18.2)	1 (20.0)
3 times/day	3 (27.3)	3 (60.0)
Single literacy item, *n* (%)		
Never	7 (63.6)	2 (40.0)
Rarely	3 (27.3)	–
Sometimes	1 (9.1)	2 (40.0)
Often	–	–
Always	–	1 (20.0)

### 3.2. Pre‐CGM Focus Groups

#### 3.2.1. Frequency of Barriers and Facilitators by SEM Level

In order of frequency, the barriers from participants from the two pre‐CGM focus groups were identified as societal‐level (155 agreements by independent reviewers; IRR = 95.0%), interpersonal/community‐level (147 agreements by independent reviewers; IRR = 89.8%), and individual‐level (150 agreements by independent reviewers; IRR = 90.9%) (Table 3). IRR calculations using the Cohen’s kappa statistic [24] revealing an average agreement of 91.9% across the three SEM levels, indicating almost perfect consistency in thematic coding between coders across the barriers. In order of frequency, the facilitators from participants were identified as societal‐level (158 agreements by independent reviewers; IRR = 96.9%), interpersonal/community‐level (151 agreements by independent reviewers; IRR = 92.1%), and individual‐level (152 agreements by independent reviewers; IRR = 93.0%) (Table 3). IRR calculations using the Cohen’s kappa statistic [24] revealed an average agreement of 94.0% across the three SEM levels, indicating almost perfect consistency in thematic coding between coders across the barriers.

**Table 3:** Pre‐focus groups agreement count and Cohen’s kappa statistic.

Level	Barriers		Facilitators	
	Agreement count	Cohen’s kappa statistic	Agreement count	Cohen’s kappa statistic
Societal	155	0.950	158	0.969
Interpersonal/community	147	0.898	151	0.921
Individual	150	0.909	152	0.930

**Figure 1:**
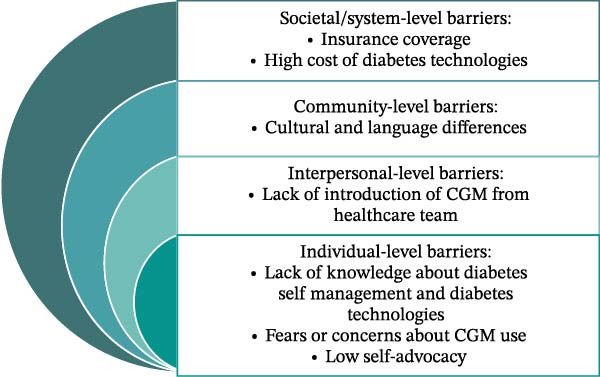
Social–ecological model and multi‐layered barriers to CGM use.

Barriers and facilitators to CGM use in the Hispanic population were identified at multiple levels using the SEM (Figure 1). At the societal and system level, barriers included limited insurance coverage and the high cost of diabetes technology, while facilitators included manufacturer‐sponsored discounts, coupons, and variable insurance benefits (Table 4). At the community and interpersonal level, concerns centered around CGM alarms, device detachment, and lack of exposure to CGM use among peers; conversely, positive word‐of‐mouth, social modeling, and caregiving support were facilitators. Interpersonally, barriers included a lack of introduction to CGM by healthcare providers and limited cultural and linguistic concordance, whereas facilitators involved strong self‐advocacy and a desire for wellness regardless of external judgment. At the individual level, challenges such as limited knowledge of diabetes technology, alarm fatigue, and fears around device use were noted; yet, motivators were the desire to reduce fingersticks, receive real‐time feedback, and improve glycemic control.

**Table 4:** Quotes regarding barriers and facilitators to CGM use prior to CGM initiation.

1. Societal	
Barriers	Insurance coverage and cost: “I’ve applied to see if I could get it, but maybe it was the insurance, they block you and they don’t give it to you. Because it’s quite… you could say it’s costly. The doctor has just prescribed the Free Style, but it’s very expensive. It’s around $45 every 14 days. So, then I have to see if I will add $45 to my list of medications, insulin, the other medication they just gave me for my stomach. It’s quite a lot.” (Interviewee <65 years). “When I tried to get the device, and this is with good insurance, I work for the university, it is still expensive.” (Interviewee <65 years) “The insurance puts up many barriers to get it approved.” (Interviewee <65 years) “Well, the difficult thing is the competition. Let there be more competition so that these insurance problems end. It’s pitiful that… imagine, people in need, more than me I think. I’m pretending that I’m fine, but people are in need or more worried than me. We come to this excellent study that you are doing, and you will realize how good it is, how necessary. And then, if they don’t get anything, it kills them. [re. You agree that the biggest barrier is insurance and cost?] Insurance and cost.” (Interviewee <65 years)
Facilitators	Manufacturer discounts: “Obviously, working for a university, I have learned of various ways to get monitors at a discount through manufacturers. Obviously, not everyone has access to computers or knows how to get in, or the indications that doctors sometimes don’t tell you as well. Look, whether you have insurance or not, you can still access and get the cards or coupons to get it at a discount, too.” (Interviewee <65 years) Insurance coverage: “[re. What makes it difficult to get one?] I wouldn’t say cost because there are insurance plans that can pay for it anyway. But I say the connection to get it.” (Interviewee >65 years)

**2. Interpersonal/community**	

Barriers	Concerns regarding alarms: “People who have a partner and you have that on, well, at night it could beep, and you are bothering the other person.” (Interviewee <65 years) CGM detachment: “I’ve heard good and bad things about them, like some of them fall off.” (Interviewee <65 years) Limited exposure to CGM: “I haven’t seen anyone wearing it. I mean, in the clinic I’ve seen people wearing it, but I haven’t asked how it is.” (Interviewee >65 years) Language‐concordant care: “The problem that I see the most, to find a doctor who speaks Spanish, and who understands the problems of Latinos. Because they are different from people of another [ethnicity]. For the food, the tradition, all that. In Florida that’s not much of a problem, because there are many Latino doctors. But in other parts of the country, it is.” (Interviewee <65 years)
Facilitators	Word of mouth and social modeling: “And the other people I’ve seen with it, which have been around 50, some of them say, “I’m thrilled, I can shower, and so on. Nothing happens. I can monitor myself with my cell phone.”” (Interviewee 1, <65 years) Hypoglycemia alerts: “My mom is 80 years old, my brother is going to St. Lucie with her, because I have my life, I’m a mom, and she says that the best thing in the world is when that beeps in the morning, because he knows that mom’s levels are low, and there he gets up to see her and can attend to her thanks to the sound that it makes. While she’s sleeping, there are alarms.” (Interviewee <65 years) Indifference to judgment: “I don’t care what others think. What concerns me is being well.” (Interviewee >65 years)

**3. Individual**	

Barriers	Alarm fatigue: “It will be beeping all day long.” (Interviewee <65 years) Limited awareness or knowledge of CGM: “It’s the first time I hear about it, I didn’t know about it, and I’ve had diabetes for years. Since I was 14.” (Interviewee <65 years) Concerns about CGM wear: “I do worry about it for bathing, sleeping, going out, certain things one does, personal, during the day. And like washing, cooking, this, that, washing your head, wetting it with shampoo. It doesn’t harm for that, right?” (Interviewee >65 years) Ability to obtain: “And how does one get that thing? How am I going to get it? To have it?” (Interviewee >65 years) Language barrier: “But not everyone has the same facility to learn a language. Maybe you learned it, I can’t learn it.” (Interviewee >65 years)
Facilitators	Fewer fingersticks: “I came to a point where I told the doctor, don’t ask me to do it [to prick my fingers], I won’t do it. I’m tired. For me, honestly, to avoid pricking my fingers, I would wear two if I want.” (Interviewee <65 years) “It would be a benefit for me and I would be very happy to wear it because there’s no one who can stand pricking their fingers. But unfortunately one has to keep pricking their fingers, but that would be an extraordinary benefit.” (Interviewee >65 years) More glucose information: “The first week will be a little hard, and you go crazy because you want to keep looking, looking, looking. You get up in the morning, it beeps, okay. If it gets too low, you need to manage it, and if it goes up, same thing. From the moment you get the hang of it in two weeks you follow your rhythm, and it is the best thing they’ve ever invented for it.” (Interviewee <65 years) Improved glycemic control: “I’m going to improve a lot with everything I’m learning, I think I can control it like that.” (Interviewee <65 years)

#### 3.2.2. Societal Barriers

At the societal level, several participants described difficulties obtaining CGMs even with insurance. Most participants expressing concerns regarding cost and insurance were under the age of 65 years. These concerns primarily focused on the high cost of the device and the complexities and barriers encountered when seeking insurance coverage for the device. One participant noted, “I’ve applied to see if I could get it, but maybe it was the insurance, they block you and they don’t give it to you… it’s quite costly” (Interviewee <65 years). Another explained, “When I tried to get the device, and this is with good insurance… it is still expensive” (Interviewee <65 years). The cumulative burden of managing and paying for multiple medications further amplified concerns around affordability. There was also frustration with systemic inefficiencies, with one participant highlighting the broader impact: “It’s pitiful… people are in need… We come to this excellent study… and then, if they don’t get anything, it kills them” (Interviewee <65 years).

#### 3.2.3. Societal Facilitators

Nevertheless, facilitators at the societal level emerged. Some participants were aware of manufacturer‐sponsored discounts and coupons, though access to these resources often required digital literacy or proactive provider guidance. As one individual shared, “Whether you have insurance or not, you can still access and get the cards or coupons… but not everyone has access to computers or knows how to get in” (Interviewee <65 years). In contrast, others with more robust insurance reported fewer financial barriers, suggesting coverage variability across plans: “I wouldn’t say cost because there are insurance plans that can pay for it anyway” (Interviewee >65 years).

#### 3.2.4. Interpersonal/Community Level Barriers

At the interpersonal and community level, several barriers were noted. Concerns about device alarms interrupting sleep or disturbing partners were common: “People who have a partner and you have that on, well, at night it could beep, and you are bothering the other person” (Interviewee <65 years). Additionally, anecdotal reports of device detachment undermined confidence: “I’ve heard good and bad things about them, like some of them fall off” (Interviewee <65 years).

Limited exposure to CGM use among peers also contributed to uncertainty: “I haven’t seen anyone wearing it… in the clinic I’ve seen people wearing it, but I haven’t asked how it is” (Interviewee >65 years). Language‐concordant care and culturally competent providers were highlighted as key unmet needs: “The problem that I see the most, to find a doctor who speaks Spanish… because they understand the problems of Latinos” (Interviewee <65 years). A sense of autonomy and self‐prioritization also emerged as a motivator, with some participants expressing indifference to external judgment: “I don’t care what others think. What concerns me is being well” (Interviewee >65 years).

#### 3.2.5. Interpersonal/Community Level Facilitators

Conversely, community‐level facilitators included positive word‐of‐mouth and social modeling. Participants primarily learned about CGMs from their community, including family members and friends. Some participants shared affirming experiences of others using CGMs comfortably, even during daily routines like showering or cooking: “And the other people I’ve seen with it, which have been around 50, some of them say, ‘I’m thrilled, I can shower, and so on. Nothing happens. I can monitor myself with my cell phone’” (Interviewee <65 years). Additionally, alarm functions were important in caregiving scenarios, particularly among families monitoring older relatives: “She says the best thing in the world is when that beeps in the morning, because he knows that mom’s levels are low… he gets up to see her” (Interviewee <65 years).

#### 3.2.6. Individual Level Barriers

At the individual level, alarm fatigue was frequently cited as a deterrent: “It will be beeping all day long” (Interviewee <65 years). Limited awareness or knowledge about CGMs was cited as a significant barrier, even among those with long‐standing diabetes: “It’s the first time I hear about it, I didn’t know about it, and I’ve had diabetes for years” (Interviewee <65 years). Participants also expressed concerns about device usability during daily activities, such as bathing or sleeping, as well as confusion over how to obtain a device or navigate eligibility. Language barriers further exacerbated access and understanding, especially for those over 65 years: “Maybe you learned [English], I can’t learn it” (Interviewee >65 years).

#### 3.2.7. Individual Level Facilitators

Despite these concerns, participants consistently identified compelling individual‐level facilitators. Chief among them was the desire to reduce or eliminate finger‐stick glucose checks. One participant stated, “For me, honestly, to avoid pricking my fingers, I would wear two if I want” (Interviewee <65 years). Others echoed similar sentiments, viewing CGM use as a major quality‐of‐life improvement.

Participants also spoke positively about the potential benefits of real‐time glucose feedback, envisioning that initial challenges would give way to improved routine and self‐management: “From the moment you get the hang of it… it is the best thing they’ve ever invented for it” (Interviewee <65 years). Ultimately, many expressed optimism about CGMs enhancing their ability to achieve better glycemic control: “I’m going to improve a lot with everything I’m learning, I think I can control it like that” (Interviewee <65 years).

### 3.3. Post‐CGM Focus Groups

Following the initial focus group discussions, participants wore a CGM for 30 days. They subsequently returned for a follow‐up focus group to reflect on their experience and evaluate the acceptability of CGM use. While a few challenges persisted—including sensors falling off, technical issues, and difficulties in carrying a phone consistently—participants overwhelmingly described the experience as beneficial. Of the 16 participants, 11 spontaneously expressed interest in continuing CGM use, though a response to the question was not required of every participant. Importantly, participants did not cite dissatisfaction with the device as their reason for not continuing. Rather, the key obstacles to sustained CGM use were insurance coverage and accessibility of obtaining devices outside of the study setting.

**Figure 2:**
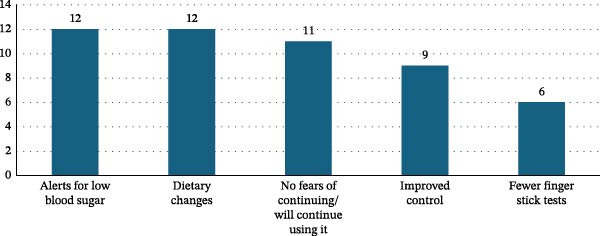
Frequency of CGM benefit themes discussed in focus groups after 30 days of CGM use in both cohorts.

Participants highlighted multiple perceived benefits (Figure 2). Many appreciated the device’s ability to track dietary impact, allowing them to make healthier food choices. As one individual described, “I like it more because it tells me which foods I eat make my sugar go up a lot. With that, I am eating better, healthier” (Interviewee <65 years). Others emphasized the value of alerts for hypoglycemia, which offered a sense of safety and anticipatory guidance: “I liked it very much—mainly the fact that it anticipated when my blood sugar would drop. It notified me” (Interviewee <65 years).

Improved glycemic control was also cited as a major advantage. Participants described how real‐time visibility into their glucose levels helped them make more informed decisions throughout the day. One participant shared, “What I loved the most was that it did keep [my blood glucose] regular, wherever I was. Because you look at it all day and you can tell, so if you are about to sit down to eat, you are going to order, you check that and you already know where it’s at” (Interviewee <65 years). Others noted that CGM use significantly reduced their need for fingerstick checks, which they found burdensome.

Participants suggested improving access to CGM for Hispanic people by introducing it early, providing more education, and addressing financial barriers. Participants reflected on the missed opportunity of not being introduced to CGM technology earlier in their diabetes journey. Several emphasized the importance of early and repeated exposure to CGM as part of routine diabetes care. One participant, living with diabetes for 6 years, expressed frustration over the lack of guidance at diagnosis: “I knew that continuous glucose monitors already existed, but the doctor did not make the effort to explain it to me clearly enough. I’m sorry—I know they are very busy… but maybe they can have an assistant, the nurse, to take two more minutes… because that alone, if he had made that effort, perhaps I would have participated a long time ago” (Interviewee <65 years). Another participant underscored the role that structured, formal education could play in demystifying CGM use, comparing it to the classes they received upon diagnosis: “There should be something so that doctors can send [patients] to a continuous monitor class… It’s different from sitting down to check it out, and to be able to ask questions, and maybe even have a trial, for 10 days. I think that would do a lot of good for the community” (Interviewee <65 years).

Cost remained a prominent barrier, particularly among participants whose insurance did not cover CGMs. Many voiced frustrations with the financial burden and the challenges of navigating discount programs. One participant shared, “I went to ask and my insurance doesn’t cover it… and you have to pay a lot to get the equipment… I always go to look for coupons, and the coupons help me a lot, it lowers the price quite a bit. I think that coupons should be provided in order to help many more people” (Interviewee <65 years). Another added that even when programs exist, access often depends on digital literacy or proactive support: “They should make it easier to access the coupons… because not everyone has access to computers or knows how to get in.”

Participants also suggested systemic improvements, such as simplifying insurance navigation and increasing the availability of Spanish‐language materials. One participant noted that they struggled to understand the device instructions due to language limitations: “I couldn’t read the directions… I tried what I could and I did, but I had to ask for help anyway, because there are things you don’t understand” (Interviewee >65 years).

By the 8‐week follow‐up call, 11 of the 16 participants had independently obtained a CGM through their provider and insurance benefits, reflecting sustained interest and a willingness to overcome barriers to continued use.

## 4. Discussion

This study highlights the multi‐level barriers and facilitators influencing CGM adoption among Hispanic individuals with insulin‐treated T2D. Using the SEM as a framework, we identified key barriers at the system, interpersonal, and individual levels, including cost and insurance challenges, lack of provider introduction to CGM, and concerns about wearing the device. After a trial period, participants expressed a strong interest in continuing CGM use, citing improved glycemic awareness, dietary modifications, and reduced reliance on fingersticks glucose monitoring as major benefits.

Our study contributes to the growing body of literature on disparities in diabetes technology use by specifically exploring barriers and facilitators to CGM adoption among Hispanic individuals with insulin‐treated T2D. The majority of our participants (54%) earned less than $25,000 per year, highlighting the financial strain many face. At the system level, insurance and cost were the top issues—especially for those under 65 years, who often had trouble getting coverage or faced high out‐of‐pocket costs. Some participants managed to get a CGM through their insurance after the study, but others ran into insurance coverage issues and expenses. These findings are consistent with previous studies that have highlighted disparities in CGM access due to insurance‐related barriers, particularly among racial and ethnic minority groups [25]. Interestingly, participants ≥65 years expressed fewer concerns about cost, suggesting that expanded insurance coverage may reduce financial barriers.

At the community and interpersonal level, participants expressed that healthcare providers often did not discuss CGM as an option. A recent study confirms low CGM prescription rates among patients with T1D and T2D in Federally Qualified Health Centers, with disparities associated with race, ethnicity, and insurance status [26]. Most participants who had learned about CGM prior to our study had primarily done so through their community, such as friends and family, not from their healthcare team. Additionally, cultural considerations emerged as a potential barrier, with participants emphasizing the importance of having Hispanic/Latino healthcare providers who understand the dietary and cultural challenges faced by Latino individuals with diabetes. The lack of Spanish‐language instructions and resources was also cited by participants, reinforcing the need for culturally tailored diabetes education.

At the individual level, barriers included concerns about device functionality, potential discomfort, and challenges in requesting CGMs from healthcare providers. These findings align with prior research by Yingling et al. [27], which identified fear, trust issues, calibration requirements, and concerns about comfort as common barriers to CGM use in individuals with T2D. Similarly, Ni et al. [28] conducted semi‐structured phone interviews with T2D patients prescribed CGM through a Medicaid program. They found that while participants initially anticipated discomfort with sensor placement due to their experience with fingersticks testing, this concern was generally overcome after initial use, a pattern also observed in our study [28].

Despite these barriers, participants also recognized the benefits of CGM technology, including improved glycemic control, greater dietary awareness, reduced need for fingersticks glucose testing, and alerts for hypoglycemia. Many participants expressed frustration that CGM had not been introduced to them earlier in their diabetes journey, suggesting that early exposure and access could improve long‐term adoption. This highlights the need to make CGM education a routine part of diabetes care for individuals who use insulin. Furthermore, efforts to improve financial accessibility—such as expanding insurance coverage, increasing provider awareness of discount programs, and advocating for systemic changes to simplify access—could help more people benefit from CGM technology.

Our study is one of the first to qualitatively explore CGM barriers among Hispanic adults with insulin‐treated T2D, using a structured framework to identify key challenges and opportunities. Strengths of the study include its focus on a diverse Hispanic population reflective of the Miami metropolitan area, the use of Spanish‐language focus groups, and the integration of pre‐ and post‐CGM use perspectives. However, limitations should be acknowledged. As a small pilot study conducted at a single center, the findings may not be generalizable to Hispanic populations in other geographic regions. While qualitative research provides in‐depth insights, it is inherently limited by cohort size and potential selection bias. Additionally, the study population had suboptimal baseline glycemic control, which may have influenced participant perspectives, including greater motivation to engage with CGM and perceive its benefits. Therefore, these findings may not fully reflect the experiences of individuals with better glycemic control or lower perceived need for diabetes technology. In addition, the transcripts were translated prior to analysis, which may have resulted in minor alterations in contextual meaning during the translation process. Future research should expand on these findings through larger, multicenter studies and quantitative analyses to further elucidate disparities in CGM adoption and develop targeted interventions.

## 5. Conclusion

Findings from this study suggest that while Hispanic individuals with insulin‐treated T2D face significant barriers to CGM access, many are willing to use CGM when provided with appropriate education and support. Addressing cost‐related barriers, increasing provider engagement in CGM discussions, improving Spanish‐language resources, and integrating structured CGM education into routine diabetes care are important steps for enhancing CGM adoption in this population.

## Author Contributions

Diana Soliman and Ashby F. Walker conceptualized and designed the study. Diana Soliman and Giuliana Arevalo collected the data. Rodolfo J. Galindo and Daniel E. Jimenez supervised the project and provided critical revisions. David A. Baidal reviewed and edited the manuscript. All authors were responsible for drafting the article and reviewing it for important intellectual content.

## Funding

Research reported in this publication was supported by the National Institute of Diabetes and Digestive and Kidney Diseases of the National Institutes of Health under Award Number P30DK111024. The content is solely the responsibility of the authors and does not necessarily represent the official views of the National Institutes of Health.

## Disclosure

All authors approved the final version to be published.

## Ethics Statement

The study protocol and consent procedures were reviewed and approved by the University of Miami Institutional Review Board (IRB #20231068). Written informed consent was obtained from all participants prior to study participation.

## Conflicts of Interest

Rodolfo J. Galindo has received research support from Novo Nordisk, Eli Lilly, and Dexcom and consulting/advisory/honoraria fees from Abbott Diabetes, AstraZeneca, Bayer, Boehringer, Dexcom, Eli Lilly, Novo Nordisk, and Medtronic. Francisco J. Pasquel reported receiving research support through the institution from Insulet, Tandem Diabetes Care, Ideal Medical Technologies, Novo Nordisk, and Dexcom; receiving consulting fees from Dexcom, and receiving consulting fees for the institution from Insulet. The other authors declare no conflicts of interest.

## Data Availability

The data that support the findings of this study are available on request from the corresponding author. The data are not publicly available due to privacy or ethical restrictions.

## References

[1] Cheng Y. J. , Kanaya A. M. , and Araneta M. R. G. , et al.Prevalence of Diabetes by Race and Ethnicity in the United States, 2011-2016, Journal of the American Medical Association. (2019) 322, no. 24, 2389–2398, 10.1001/jama.2019.19365. 31860047 PMC6990660

[2] Kirk J. K. , Passmore L. V. , and Bell R. A. , et al.Disparities in A1C Levels Between Hispanic and Non-Hispanic White Adults With Diabetes: A Meta-Analysis, Diabetes Care. (2008) 31, no. 2, 240–246, 10.2337/dc07-0382, 2-s2.0-38949155212. 17977939

[3] Conway R. B. , Gerard Gonzalez A. , and Shah V. N. , et al.Racial Disparities in Diabetes Technology Adoption and Their Association With HbA1c and Diabetic Ketoacidosis, Diabetes, Metabolic Syndrome and Obesity. (2023) 16, 2295–2310, 10.2147/DMSO.S416192. PMC10404403 37551339

[4] Haw J. S. , Shah M. , and Turbow S. , et al.Diabetes Complications in Racial and Ethnic Minority Populations in the USA, Current Diabetes Reports. (2021) 21, no. 1, 2–20, 10.1007/s11892-020-01369-x. 33420878 PMC7935471

[5] Galindo R. J. and Aleppo G. , Continuous Glucose Monitoring: The Achievement of 100 Years of Innovation in Diabetes Technology, Diabetes Research and Clinical Practice. (2020) 170, 10.1016/j.diabres.2020.108502, 108502. 33065179 PMC7736459

[6] Litchman M. L. , Ng A. , and Sanchez-Birkhead A. , et al.Combining CGM and an Online Peer Support Community for Hispanic Adults With T2D: A Feasibility Study, Journal of Diabetes Science and Technology. (2022) 16, no. 4, 866–873, 10.1177/19322968211032278. 34414787 PMC9264448

[7] Lind N. , Christensen M. B. , and Hansen D. L. , et al.Comparing Continuous Glucose Monitoring and Blood Glucose Monitoring in Adults With Inadequately Controlled, Insulin-Treated Type 2 Diabetes (Steno2Tech Study): A. 12-Month, Single-Center, Randomized Controlled Trial, Diabetes Care. (2024) 47, no. 5, 881–889, 10.2337/dc23-2194. 38489032

[8] Reaven P. D. , Newell M. , and Rivas S. , et al.Initiation of Continuous Glucose Monitoring Is Linked to Improved Glycemic Control and Fewer Clinical Events in Type 1 and Type 2 Diabetes in the Veterans Health Administration, Diabetes Care. (2023) 46, no. 4, 854–863, 10.2337/dc22-2189. 36807492 PMC10260873

[9] ElSayed N. A. , Aleppo G. , and Bannuru R. R. , et al.7. Diabetes Technology: *Standards of Care in Diabetes—2024* , Diabetes Care. (2024) 47, S126–S144, 10.2337/dc24-S007. 38078575 PMC10725813

[10] Grunberger G. , Sherr J. , and Allende M. , et al.American Association of Clinical Endocrinology Clinical Practice Guideline: The Use of Advanced Technology in the Management of Persons With Diabetes Mellitus, Endocrine Practice. (2021) 27, no. 6, 505–537, 10.1016/j.eprac.2021.04.008. 34116789

[11] Agarwal S. , Schechter C. , and Gonzalez J. , et al.Racial-Ethnic Disparities in Diabetes Technology Use Among Young Adults With Type 1 Diabetes, Diabetes Technology & Therapeutics. (2021) 23, no. 4, 306–313, 10.1089/dia.2020.0338. 33155826 PMC7994432

[12] Agarwal S. , Kanapka L. G. , and Raymond J. K. , et al.Racial-Ethnic Inequity in Young Adults With Type 1 Diabetes, The Journal of Clinical Endocrinology and Metabolism. (2020) 105, no. 8, e2960–e2969, 10.1210/clinem/dgaa236. 32382736 PMC7457963

[13] Fantasia K. L. , Wirunsawanya K. , and Lee C. , et al.Racial Disparities in Diabetes Technology Use and Outcomes in Type 1 Diabetes in a Safety-Net Hospital, Journal of Diabetes Science and Technology. (2021) 15, no. 5, 1010–1017, 10.1177/1932296821995810. 33719610 PMC8442173

[14] Ebekozien O. , Mungmode A. , and Sanchez J. , et al.Longitudinal Trends in Glycemic Outcomes and Technology Use for Over 48,000 People With Type 1 Diabetes (2016-2022) From the T1D Exchange Quality Improvement Collaborative, Diabetes Technology & Therapeutics. (2023) 25, no. 11, 765–773, 10.1089/dia.2023.0320. 37768677

[15] Agarwal S. , Simmonds I. , and Myers A. K. , The Use of Diabetes Technology to Address Inequity in Health Outcomes: Limitations and Opportunities, Current Diabetes Reports. (2022) 22, no. 7, 275–281, 10.1007/s11892-022-01470-3. 35648277 PMC9157044

[16] Kanbour S. , Jones M. , and Abusamaan M. S. , et al.Racial Disparities in Access and Use of Diabetes Technology Among Adult Patients With Type 1 Diabetes in a U.S. Academic Medical Center, Diabetes Care. (2023) 46, no. 1, 56–64, 10.2337/dc22-1055. 36378855 PMC9797654

[17] Odugbesan O. , Addala A. , and Nelson G. , et al.Implicit Racial-Ethnic and Insurance-Mediated Bias to Recommending Diabetes Technology: Insights From T1D Exchange Multicenter Pediatric and Adult Diabetes Provider Cohort, Diabetes Technology & Therapeutics. (2022) 24, no. 9, 619–627, 10.1089/dia.2022.0042. 35604789 PMC9422789

[18] Walker A. F. , Hood K. K. , and Gurka M. J. , et al.Barriers to Technology Use and Endocrinology Care for Underserved Communities With Type 1 Diabetes, Diabetes Care. (2021) 44, no. 7, 1480–1490, 10.2337/dc20-2753. 34001535 PMC8323174

[19] Agarwal S. , Crespo-Ramos G. , and Long J. A. , et al.I Didn’t Really Have a Choice": Qualitative Analysis of Racial-Ethnic Disparities in Diabetes Technology Use Among Young Adults With Type 1 Diabetes, Diabetes Technology & Therapeutics. (2021) 23, no. 9, 616–622, 10.1089/dia.2021.0075. 33761284 PMC8501459

[20] Been R. A. , Lameijer A. , and Gans R. O. B. , et al.The Impact of Socioeconomic Factors, Social Determinants, and Ethnicity on the Utilization of Glucose Sensor Technology Among Persons With Diabetes Mellitus: A Narrative Review, Therapeutic Advances in Endocrinology and Metabolism. (2024) 15, 10.1177/20420188241236289, 20240311. PMC10929059 38476216

[21] Bronfenbrenner U. , Cambridge, MA and London, 1979, Harvard University Press.

[22] Damschroder L. J. , Reardon C. M. , and Widerquist M. A. O. , et al.The Updated Consolidated Framework for Implementation Research Based on User Feedback, Implementation Science. (2022) 17, no. 1, 17–75, 10.1186/s13012-022-01245-0. 36309746 PMC9617234

[23] Onwuegbuzie A. J. , Dickinson W. B. , and Leech N. L. , et al.A Qualitative Framework for Collecting and Analyzing Data in Focus Group Research, International Journal of Qualitative Methods. (2009) 8, no. 3, 1–21, 10.1177/160940690900800301.

[24] McHugh M. L. , Interrater Reliability: The Kappa Statistic, Biochemia Medica. (2012) 22, 276–282, 10.11613/BM.2012.031. 23092060 PMC3900052

[25] Alkabbani W. , Cromer S. J. , and Kim D. H. , et al.Overall Uptake and Racial, Ethnic, and Socioeconomic Disparities in the Use of Continuous Glucose Monitoring Devices Among Insulin-Treated Older Adults With Type 2 Diabetes, Diabetes Care. (2025) 48, no. 8, 1377–1385, 10.2337/dca25-0006. 40445849 PMC12718080

[26] Wallia A. , Agarwal S. , and Owen A. L. , et al.Disparities in Continuous Glucose Monitoring Among Patients Receiving Care in Federally Qualified Health Centers, JAMA Network Open. (2024) 7, no. 11, 10.1001/jamanetworkopen.2024.45316, e2445316. 39576644 PMC11584923

[27] Yingling L. , Allen N. A. , and Litchman M. L. , et al.An Evaluation of Digital Health Tools for Diabetes Self-Management in Hispanic Adults: Exploratory Study, JMIR Diabetes. (2019) 4, no. 3, 10.2196/12936, 2-s2.0-85071372109, e12936. 31313657 PMC6664655

[28] Ni K. , Tampe C. A. , and Sol K. , et al.Continuous Glucose Monitor: Reclaiming Type 2 Diabetes Self-Efficacy and Mitigating Disparities, Journal of the Endocrine Society. (2024) 8, no. 8, 10.1210/jendso/bvae125, bvae125. 38974988 PMC11223994

